# The Somali Distress and Resilience Scale: Development of a novel measure for Somali adults

**DOI:** 10.1177/13634615241272982

**Published:** 2024-08-31

**Authors:** Alec Terrana, William Bruno, Najla Ibrahim, Bonnie N. Kaiser, Jenny Wei, Wael Al-Delaimy

**Affiliations:** 1 School of Medicine, 8784 University of California San Diego, La Jolla, CA, USA; 2 Department of Emergency Medicine, 8784 University of California San Diego, San Diego, CA, USA; 3 Somali Family Service of San Diego, San Diego, CA, USA; 4 Department of Anthropology and Global Health Program, 8784 University of California San Diego, San Diego, CA, USA; 5 Herbert Wertheim School of Public Health, 8784 University of California San Diego, San Diego, CA, USA; 6 School of Medicine, 8784 University of California San Diego, San Diego, CA, USA

**Keywords:** mental health, qualitative, refugee, resilience, scale development, Somali

## Abstract

Although resilience has been identified as an important mediator of negative mental health outcomes among refugee populations, there are few culturally specific measures of resilience among such communities and no such measure among Somalis. In this study we aimed to develop a culturally appropriate measure of resilience specific to Somali adults in San Diego, as an example of a vulnerable refugee community. A community-based, exploratory sequential mixed method investigation was conducted via focus group discussions (*n* = 4), cognitive interviews (*n* = 4), and iterative survey adaptation. Somali refugee adults in San Diego (*N* = 183) were surveyed with this novel scale, a standardized measure of resilience, and assessments of depression, anxiety, and PTSD. Results were analyzed via correlation coefficients and multivariate linear regression modeling. Qualitative findings supported the inclusion of items addressing both barriers and facilitators of good mental health, which resulted in the development of the Somali Distress and Resilience Survey (SDRS). Linear regression analysis revealed that the SDRS demonstrated significant associations with symptoms of depression and PTSD, while the standardized measure of resilience did not demonstrate associations with any of the mental health outcomes assessed. The SDRS identified obstacles to resilience among Somali individuals, placing them at risk of developing negative mental health outcomes. Our novel measure also demonstrated more robust relationships with these outcomes than a standardized measure of resilience, suggesting greater utility of the adapted scale. However, the SDRS's development raises larger questions about the limitations of developing and comprehensively evaluating novel resilience measures in a community-based setting.

## Background

### Mental health within the Somali diaspora

Somalia has experienced a long history of political conflict, extending back to General Mahammad Siad Barre's establishment of a military dictatorship in 1969 and continuing through the civil war following his overthrow, which persisted alongside UN-sponsored humanitarian aid and military support (Tikkanen, 2022). Somalia's instability has been compounded by foreign military interventions, famine, and drought, and it is estimated that nearly 45% of the population has been displaced beyond Somalia's borders—placing the nation as the world's fourth highest source country for refugees after only Syria, Afghanistan, and Ukraine (CIA World Factbook, 2023; Metz, 1993; Sperber, 2019). Somali immigrants and refugees have been arriving in the United States since the early 1990s, with Minneapolis–St Paul, Minnesota as the most common area of resettlement and San Diego, California the second largest (Burks, 2013; CDC, 2018).

Somali refugees in the United States face a multitude of adversities. In addition to trauma exposure due to the civil war and subsequent displacement, studies have also identified the dramatic psychological toll of acculturation challenges (Ellis et al., 2008, 2013). Among these difficulties of acculturation are discrimination and racism; social isolation; insufficient access to fair employment and affordable housing; and a healthcare system that often feels inaccessible due to its lack of recognition of and respect for Somali beliefs and practices (Betancourt et al., 2015; Ellis et al., 2013, 2016; Streuli et al., 2021; Terrana et al., 2022; Tiilikainen & Koehn, 2011). These challenges have been exacerbated by a fracturing of the familial unit due to deaths and displacement, as well as role distortion within existing families, which is often due to changes in power dynamics between parents and children relating to their respective abilities to navigate language barriers (Schuchman & McDonald, 2008; Scuglik et al., 2007).

### Culturally attuned measures of mental health

Accurately assessing the psychological toll of these adversities among members of the Somali diaspora is challenging, in part due to the unclear validity of applying a standardized measure to communities with a cultural background different from that of the population in which it was originally developed (Kaiser et al., 2022; Kohrt & Kaiser, 2021; Van Ommeren et al., 1999). This lack of culturally appropriate mental health assessment tools remains a significant barrier to effective screening, not only among Somalis, but across many populations outside the Global North. Direct translation of existing instruments is likely to produce misleading results due to breakdowns of conceptual equivalence, and it is therefore unsurprising that measures subjected to rigorous cultural adaptation have been shown to function better in validation studies (Ali et al., 2016; Weobong et al., 2009). Consequently, there have been increasing demands for culturally adapted measures of mental health for both research and clinical use (Atilola, 2015; Fennig, 2024; Kaiser et al., 2013; Kohrt et al., 2011).

### Resilience as a promotor of mental health

Operationalizing “resilience” has remained inconsistent since the construct gained prominence in mental health literature in the 1970s. Researchers have noted that, among the diversity of definitions, there is agreement that resilience entails (a) positive adaptation and good mental health (b) despite exposure to significant adversity (Luthar et al., 2000). Contemporary conceptions of resilience also prioritize the socio-ecological dimensions of the construct, which account for the various spheres of influence that act upon an individual, including their family; educational and employment environment; political and economic systems; and the broader cultural norms, values, and beliefs (Betancourt & Khan, 2008).

This inherently contextual, socio-ecological nature of resilience has called into question the use of standardized measures of resilience, which tend to operationalize it as an individual capacity or trait like “grit” (Kim et al., 2019; Panter-Brick et al., 2018). Although a number of standardized measures, such as the Connor-Davidson Resilience Scale (CD-RISC) and the Child Youth and Resiliency Measure (CYRM), have been subjected to rigorous field testing in a variety of populations and cultural contexts, there is no gold standard (Terrana & Al-Delaimy, 2023). This recognition has prompted calls for a mixed methods approach to developing resilience measures, starting with qualitative fieldwork for understanding concepts of resilience in a particular cultural context in order to justify the inclusion and appropriate translation of any individual items on the scale, along the lines of what is done for mental health assessment tools (Fennig, 2024; Kaiser & Weaver, 2019; Snodgrass et al., 2023; Weaver & Kaiser, 2015). While such an approach remains the exception within the field, adopting mixed methods to develop locally-contextualized resilience surveys is even less common than those assessing negative mental health outcomes, such as anxiety and depression, with few published models of this approach (Kim et al., 2019; Panter-Brick et al., 2018). This relative dearth of resilience measures attuned to local context points to an area worthy of further investigation, not only for contributing the scale itself to the literature, but also for extracting general principles that can support future development of ethnographically-grounded, strengths-based measures.

### The present study

This study aimed to develop a novel measure, the Somali Distress and Resilience Scale (SDRS), that assesses current mental health status and resilience in alignment with the experiences of members of the Somali community in San Diego, California. We also aimed to demonstrate a process for local development and testing of a resilience scale, by combining the strengths of qualitative and ethnographic studies characterizing resilience in socio-ecological contexts, alongside literature on local scale development for mental health. This article builds upon our prior qualitative explorations of resilience among Somalis, applying these findings to the development and initial testing of a quantitative scale (Terrana et al., 2022).

Our primary aim was to develop an instrument that can accurately capture the construct of resilience among members of the Somali diaspora in the United States, a culturally unique and vulnerable population. This instrument would identify individuals with limited resilience resources who might be vulnerable to mental illnesses, as well as identify resilient resources that protect against developing negative mental health outcomes and can inform the implementation of potential interventions. We are not aware of any resilience scale developed or adapted for Somalis. Given the paucity of studies documenting the mixed methods development of culturally sensitive resilience surveys, we adopted a primarily exploratory approach that outlines the development and assessment process, as well as key hurdles and tensions inherent to this work, as a response to the Euro-American centric bias inherent to most resilience scales (Fennig, 2024; Kaiser & Weaver, 2019; Kim et al., 2019; Mendenhall & Kim, 2019).

## Methods

To conduct a project that was most in line with the perspectives and needs of members of San Diego's Somali community, researchers at University of California San Diego (UCSD) adopted a community-engaged collaborative approach through close partnership with Somali Family Service of San Diego (SFS), a community-based social service organization that has been providing culturally and linguistically appropriate programs to the Somali community of San Diego since 2000. SFS's Director of Health & Wellness, community health workers (CHW), and researchers from UCSD collaborated closely to create the overarching research plan, develop qualitative and quantitative data collection materials, recruit participants, and interpret results through an iterative process that took cultural and linguistic considerations into account.

### Prior qualitative study

The present effort to construct and preliminarily assess a novel resilience measure was preceded by a qualitative study that adopted ethnographic methods to determine which themes would be most relevant to evaluating resilience among Somalis in San Diego (Authors, 2022). Qualitative data were collected through focus group discussions (FGDs) between July and August 2020 via Zoom video conferencing and were facilitated by two co-investigators, one from UCSD and one from SFS. FGDs were guided by a series of open-ended questions on the topics of life pressures and concerns, sources of physical or emotional distress, and coping mechanisms and supportive resources. This FGD guide was iteratively updated to focus more closely on areas of particular interest or relevance to the research aims and data collection continued until we had determined that we had achieved thematic saturation.

FGD data were analyzed using a qualitative content analysis approach in Dedoose (Hsieh & Shannon, 2005; SocioCultural Research Consultants, 2021). The lead qualitative analyst from UCSD conducted a preliminary round of open coding of the data from the first FGD to develop an initial codebook that identified all potential themes that emerged, which was then modified through a secondary, more focused round of coding to designate themes as dominant or subordinate (Gibson & Brown, 2009). A team member from SFS then reviewed the codebook to clarify and confirm the thematic relationships and a sub-set of initial code assignments were analyzed by an additional coder with no prior familiarity with the project, which demonstrated “excellent agreement” of inter-rater reliability with a Cohen's kappa of 0.795 (Cohen, 1960; Fleiss, 1971; Authors, 2022).

Focus group findings suggested that resilience among members of San Diego's Somali community is rooted in supportive factors and access to resources that promote positive coping skills in the face of acculturation barriers. Participants identified the interrelated experiences of resource hardships and discrimination due to racism, Islamophobia, and xenophobia as the primary barriers to well-being that resilience would help them overcome. Conversely, participants identified a rich range of resources that constituted resilience and a positive approach to adversity. These include a collective identity of Somalis as survivors due to their shared history of overcoming adversity, the Muslim faith and its promotion of accepting whatever challenges might come your way due to the support of Allah, and a strong sense of communal oneness (*walaal-nimo*) that encourages reciprocal helping behaviors. For further detail on our qualitative methods and results, please refer to the published findings on this aspect of the larger study (Authors, 2022).

### Participant characteristics and setting

Participants were recruited to the present study by team members from SFS through social media, flyers, and word-of-mouth communication. Inclusion criteria included being a Somali refugee or immigrant over the age of 18 living in San Diego County who speaks English and was willing and able to participate in focus groups, group cognitive interviews, or surveys. Participants were given $30 for their participation in a focus group or cognitive interview and $25 for completing the resilience survey. The study was approved by the UCSD Institutional Review Board.

### Procedure

This study was designed according to established methods for transcultural adaptation of assessment tools and development of novel mental health instruments (Kaiser & Weaver, 2019; Snodgrass et al., 2023; Weaver & Kaiser, 2015). This approach was intended to both elicit aspects of resilience that accurately reflected the perspectives of study participants and to maintain an equivalence of meaning in the translation of qualitative data to a quantitative measure.

#### Scale development.

The emergent themes from qualitative analysis directly informed the development of a set of 19 preliminary items for the resilience scale (e.g., “No matter the challenges I face, I have the patience (*sabr*) to make it over and go on;” “I participate in organized religious activities”). Each item was considered by the research team, which consisted of the FGD facilitators from both UCSD and SFS, as well as an expert in the development and cross-cultural adaptation of psychological scales based on qualitative data. Team members discussed how items should be worded based on how themes had initially emerged in the FGDs so that they would be most comprehensible to survey participants. Items were incorporated into the scale by consensus, with preference given to the viewpoint of the Somali co-investigator in the case of difficulty in achieving full agreement. Following completion of the preliminary set of items, FGD transcripts were then reviewed again to identify any explicitly stated or implied problems with the items to improve the comprehensibility and relevance of the items.

#### Pilot testing via cognitive interviewing and item adaptation.

The preliminary survey was piloted via cognitive interviewing over a password-protected Zoom session in September 2020. Due to scheduling limitations, group interviews were conducted with four SFS staff members who had no prior involvement in the study and were identified as key informants within the Somali community who would have a unique ability to speak broadly to the experiences of their fellow community members. Preliminary survey items were presented exclusively in English.

Participants were asked to respond to each item of the measure one at a time and, after having responded, were then asked how they understood the item and to describe their decision-making process when giving their answer. Specifically, participants were asked “Why did you give that response?” and “How did you understand that question?” Prior research has shown the value of this approach through the participant's verbalization of their interpretation of each item to indicate which of these items might be interpreted differently than intended and thus require adaptation (Kaiser et al., 2019). Cognitive interview findings informed a round of revisions to further improve comprehensibility and reduce redundancy. For example, given the centrality of Islam to many Somalis’ lives, items regarding access to social and material support were specifically reframed with the caveat, “Other than God,” to assess access to resources within the community in contrast to the items more specifically addressing the role of faith (“I draw strength/support from God”).

#### Quantitative data collection.

The Somali Distress and Resilience Scale (SDRS) was piloted among a sample of 183 Somali adults, along with a suite of mental health assessment tools: a subset of items from the Harvard Trauma Questionnaire (HTQ), which focused solely on questions related to post-traumatic stress disorder (HTQ-PTSD); the 2-item Patient Health Questionnaire (PHQ-2), a screening tool for depression; and the 2-item Generalized Anxiety Disorder Assessment (GAD-2), a screening tool for generalized anxiety disorder (Berthold et al., 2019; Kroenke et al., 2003, 2007). While the PHQ was chosen due to the prior adaptation and validation of the PHQ-9 among Somalis, the GAD-2 was selected for its brevity and widespread usage, given the absence of an anxiety measure adapted for use among Somalis (Bhui et al., 2006; Nallusamy et al., 2016). The Connor-Davidson Resilience Scale (CD-RISC) was chosen as the standardized resilience measure for comparison due to its highly rated psychometric properties in U.S. samples. It was co-administered without any adaptation to a subset of participants (*n* = 48) as a comparator with the SDRS through assessing their relative strength of associations with the PHQ and GAD (Windle et al., 2011).

All surveys were made available in English for digital dissemination via SFS's social media accounts beginning in March 2021, with the intention of recruiting at least 200 participants. Hard copies were also produced for in-person distribution by SFS's CHWs, who provided translation services, as needed. Round one of data collection (SDRS, HTQ-PTSD, PHQ, GAD) spanned a period of 8 months, concluding in October 2021, while round two of data collection (CD-RISC, repeat SDRS, from a subset of participants) occurred from May to June 2022.

#### Quantitative data analysis.

PTSD symptom severity was computed by averaging responses on the 16 PTSD items from the HTQ, with potential scores ranging from 1–4. Depression and anxiety were assessed via summed scores of the PHQ-2 and GAD-2, respectively, with potential scores ranging from 0–6 on each. HTQ-PTSD, PHQ-2, and GAD-2 scores were also summed to determine an overall mental health symptom burden score, ranging from 1–16. Resilience scores (SDRS-R) were calculated as the sum of the 11 SDRS items assessing resilience, with one item reversed-scored so that a higher score indicates higher resilience, with potential scores ranging from 11–55. A mental health symptom score (SDRS-MH) was derived from the sum of the four SDRS items assessing mental health symptoms, with potential scores ranging from 4–20. Lastly, the CD-RISC consisted of 25 items, evaluated on a 5-point Likert scale, with scores summed to create a total score ranging from 0–100.

Correlation between SDRS-R score and scores on the PHQ-2, GAD-2, and HTQ-PTSD scores were analyzed using a Spearman's rank correlation coefficient as a non-parametric correlation measure because the data was not normally distributed. Linear regression was also used to develop multivariable models that adjusted for age and sex while evaluating SDRS-R score, treated as a continuous independent variable, as a predictor of scores on the PHQ-2, GAD-2, and HTQ-PTSD, also treated as continuous variables. Linear regression analyses were also conducted for the subset of participants that were administered the CD-RISC, examining associations between either SDRS-R or CD-RISC score and SDRS-MH, PHQ-2, GAD-2, and HTQ-PTSD. Correlation between the SDRS-R and the CD-RISC was also analyzed using a Spearman's rank correlation coefficient. Finally, a paired *t*-test was used to assess for differences in SDRS-R score between rounds of data collection.

## Results

### Scale development and cognitive interview results

Elements of resilience identified in qualitative data were distilled into a series of 19 statements assessing resilience (Appendix 1). Cognitive interview participants advocated for items assessing anxiety and depression to be added to the survey and asked in ways most immediately understood by Somalis: as somatic complaints. These include difficulty sleeping, loss of appetite, and “worrying too much,” each of which was incorporated into the scale as an internal measure of the states against which resilience presumably protects. Their inclusion was intended to capture the most salient indicators of poor mental health, and therefore low resilience, among Somalis that might not otherwise be accounted for by non-culturally adapted measures of anxiety and depression.

Participants also advised against using items that would be likely to be unanimously affirmed or denied based on alignment with core cultural values and expectations (cultural scripts) in the formal scoring process, such as, “I feel a sense of oneness (*walaal-nimo*) with the broader Somali community,” and, “The Somali people are able to survive any challenges they face.” This was based on the belief that such items would not allow for meaningfully differentiating among individuals. However, given the centrality of these topics, cognitive interviewing participants also noted that survey respondents would find the complete absence of these items to be conspicuous. As such, they recommended including them as unscored items that would give respondents an opportunity to address these essential areas (items 16 and 17). For Item 16, results indicate that most participants placed the greatest importance on “Belief in the afterlife (“akhirah”)” and “Knowing that God (“Allah”) will support me” as areas that they draw strength from, while “Feeling hope that tomorrow will be better” and “An individual who I look up to as a role model” were least important. For Item 17, close to half the participants felt that the greatest challenge to their happiness and sense of well-being comes from “the way I am treated because of my race and/or religion.”

Cognitive interviewing also affirmed the necessity of scale brevity and minimizing survey burden, which were highlighted as priorities not only for the resilience survey, but also for the overall bundle of surveys. This resulted in removal of items determined to be redundant or poor proxies for the underlying concept of interest, as in the case of “I cry easily” being used as an analog for sadness in the subscale assessing anxiety and depression. This process of pilot testing and subsequent adaptations resulted in the finalized SDRS with 15 scored items, comprised of a subscale evaluating symptoms of poor mental health (SDRS-Mental Health (SDRS-MH), items 1–4) and a subscale evaluating resilience (SDRS-Resilience (SDRS-R), items 5–15) (Tables 1 and 2).

**Table 1.** Final Somali Distress and Resilience Survey items.

Scored items (1–15)
Somali Distress and Resilience Survey – Mental Health (SDRS-MH) (1–4)
The following statements address how you’ve been feeling physically and emotionally over the past month:
1. I have difficulty falling asleep or staying asleep.
2. I do not have an appetite.
3. I worry too much about things.
4. There have been situations that have made me feel sad.
Somali Distress and Resilience Survey – Resilience (SDRS-R) (5–15)
The following statements address your relationships with others and the larger community over the past month:
5. I have supported someone in need.
6. I draw strength/support from God.
7. Other than God, there is at least one person in my life that I trust and can talk to during difficult or stressful times.
8. Other than God, there is at least one person in my life that I can trust to give me good advice when I have a problem.
9. Other than God, there is at least one person in my life who can offer me support in finding a job, housing, or transportation if I need it.
10. I have lost opportunities for job placement and income because of language or cultural barriers as a member of the Somali community. (reverse-scored)
The following statements address how you approach challenges in your life:
11. Overcoming challenges in the past gives me confidence in dealing with new challenges and difficulties.
12. I try to see the humorous side of things when I am faced with problems.
13. Under pressure, I stay focused and think clearly.
14. I prefer to take the lead in solving problems rather than letting others make all the decisions.
15. I can make unpopular or difficult decisions that affect other people, if it is necessary.

Unscored Items (16–18)
16. Rank the importance of the following areas that you draw strength from, with 1 being the most important and 7 being the least important. Please only use each ranking once. Belief in the afterlife (*akhirah*). An individual (including historical figures) who I look up to as a role model. My sense of oneness (*walaal-nimo*) with the Somali community. Prayer and/or religious rituals. Having a sense of control over my life. Knowing that God (“Allah”) will support me. Feeling hope that tomorrow will be better.
17. Rank the importance of the following areas that challenge your happiness and sense of well-being, with 1 being the biggest challenge and 7 being the least challenging. Please only use each ranking once. The way I am treated because of my race and/or my religion. Access to opportunities for schooling or job training that meet my needs. Difficulties of connecting with my faith and/or God (*Allah*). The availability of affordable housing. My ability to form relationships with members of my community. Language barriers I face on a daily basis. My ability to see doctors or nurses who understand my unique needs as a Somali.
18. Do you have any additional thoughts or comments about your experience with facing challenges or the questions asked here?

**Table 2.** Participant demographics.

Characteristics	Overall (*n* = 176)	SDRS-R Score Mean (SD)	*p*-value using ANOVA
Age, years: Mean (*SD*)	32.4 (15.4)	2.6 (0.6)	.16
Gender, *n* (% or *SD*)			
Male	49 (27.8)	2.5 (1.13)	.72
Female	125 (71.6)	2.6 (0.79)	
Unknown	1 (0.6)	3 (−)	
Education, *n* (% or *SD*)			
Less than High School	33 (18.8)	2.4 (1.8)	.46
High School	65 (36.9)	2.6 (1.0)	
Bachelor's Degree	41 (23.3)	2.7 (1.43)	
Bachelor's Degree +	34 (19.3)	2.6 (1.1)	
Unknown	3 (1.7)	2.3 (3.2)	
Years lived in the United States, *n* (%)			
1–10 Years	35 (19.9)	2.3 (1.6)	.02
10+ Years	137 (77.8)	2.6 (0.7)	
Unknown	4 (2.3)	2.6 (2.86)	
HTQ-PTSD score, mean (*SD*)	1.6 (0.54)		
PHQ-2 Depression score, mean (*SD*)	1.3 (1.3)		
GAD-2 Anxiety score, mean (*SD*)	1.5 (1.3)		

### Quantitative results

#### Demographic characteristics of participants.

Of the 183 participants who completed the SDRS, seven were excluded due to insufficient data, leaving 176 participants for analysis. Overall, 71.6% were females. Ages ranged from 18 to 91 (*M* = 32.4, *SD* = 15.4); the vast majority received high school education or higher; and a great majority lived in the United States for more than 10 years (77.8%). Overall mean HTQ-PTSD score was 1.6, mean PHQ-2 depression score was 1.3, and mean GAD-2 anxiety score was 1.5.

To assess internal consistency, alpha reliability coefficients were run for each scale. The results were as follows: HTQ-PTSD (α = .94), PHQ-2 (α = .70), GAD-2 (α = .75), CD-RISC, (α = .81), SDRS-MH (α = .84), SDRS-R (α = .81).

#### SDRS associations.

Based on Spearman's correlation coefficients, the SDRS-R demonstrated low to moderate inverse correlations with PHQ-2 score (ρ = −0.33, *p* < .01), GAD-2 score (ρ = −0.23, *p* < .01), and HTQ-PTSD score (ρ = −0.30, *p* < .01) (Table 3).

**Table 3.** Spearman's correlation coefficients among scales.

	PHQ-2	GAD-2	HTQ-PTSD	SDRS-MH
SDRS-R (*n* = 168)	−0.33**	−0.23**	−0.30**	−0.21**
SDRS-MH (*n* = 174)	0.62**	0.53**	0.61**	
CD-RISC (*n* = 44)	0.19	−0.047	0.15	0.014

*p < .05; **p < .01.

After adjusting for age and sex using a multivariable linear regression model, SDRS-R score continued to demonstrate a significant association with the PHQ-2 (β = −.43, *p* < .01) and the HTQ-PTSD (β = −.20, *p* < .01) but not with the GAD-2 (β = −.23, *p* = .143) (Table 4).

**Table 4.** Regression models treating resilience scales as predictors and mental health symptom scales as outcomes.

	PHQ-2					GAD-2					HTQ-PTSD					SDRS-MH				
	β Est.	*SE*	95% CI		*p*	β Est.	*SE*	95% CI		*p*	β Est.	*SE*	95% CI		*p*	β Est.	*SE*	95% CI		*p*
			*LL*	*UL*				*LL*	*UL*				*LL*	*UL*				*LL*	*UL*	
SDRS-R (*n* = 168)	−.43	.15	−.73	−.14	.004	−.23	.15	−.54	.075	.14	−.20	.061	−.32	−.077	.001	−.18	.83	−.39	.024	.083
CD-RISC (*n* = 44)	.013	.01	−.0078	.034	.21	.003	.015	−.027	.033	.83	.007	.005	.003	.017	.169	.0053	.0087	−.012	.023	.57

*Note.* All models control for age and sex.

Within the subset of participants who completed the CD-RISC and provided sufficient data for analysis (*n* = 44), the mean CD-RISC score was 71 (out of 100). A paired-sample *t*-test indicated no significant difference between SDRS-R scores between the two time points of data collection, allowing for comparison of the CD-RISC and participants’ initially reported mental health scores. CD-RISC and SDRS-R were found to be significantly correlated via Spearman's correlation (ρ = 0.55, *p* < .01). No significant relationship was found between CD-RISC score and mental health outcomes as assessed via the PHQ-2 (ρ = 0.19, *p* = .22), GAD-2 (ρ = −0.047, *p* = .76), or HTQ-PTSD (ρ = 0.15, *p* = .32) (Table 3). Further analysis via linear regression did not result in any of these associations becoming significant (Table 4).

## Discussion

Our study used mixed methods to develop and pilot a novel, culturally informed measure of resilience among Somali immigrants. Based on qualitative data, resilience constituted access to culturally meaningful support systems and coping skills, as well as items reflecting optimistic or determined attitudes that most resilience scales measure. The resilience sub-scale (SDRS-R) was negatively correlated with all mental health outcomes assessed (depression, anxiety, PTSD), and all associations remained significant after adjusting for demographic characteristics, except with anxiety. These results are consistent with the theoretical model of resilience as a protective effect against mental illness symptoms, though we cannot speak to causality in our study. The mental health sub-scale (SDRS-MH), which was developed due to community members' explicit requests to address mental health concerns within a resilience measure, also demonstrated the expected associations. This scale was significantly and inversely associated with resilience, demonstrating internal validity and potential for future use in these communities as an alternative to standardized measures of mental health.

In contrast, the standardized measure of resilience (CD-RISC) used in our study failed to demonstrate any significant association with symptoms of anxiety, depression, or PTSD, either in bivariate correlations or within a multivariable model adjusting for demographic factors. While this lack of significant association might be attributable to the small sample size that received both the SDRS and the CD-RISC, the correlation coefficients were very low. The culturally-tailored SDRS-R thus seemingly demonstrates its value over a standardized measure when assessing Somali individuals’ risk for developing negative mental health outcomes, suggesting utility in identifying low resilience individuals who might benefit from proactive intervention. For example, organizations like SFS could use the SDRS-R to identify community members who would most benefit from active connection to community supports and resources, perhaps through their existing CHW network. Items 16 and 17, which assess the relative importance of specific supportive factors and barriers to well-being, respectively, can help guide these preventive measures by offering insight into the specific areas that each survey respondent would benefit from strengthening, thus allowing for more targeted interventions.

The fact that the SDRS-R demonstrated stronger associations with negative mental health outcomes in contrast to the standardized measure of resilience contributes to existing literature on the benefits of developing mental health instruments that are aligned with the target population's explanatory models and unique conceptualization of the construct in question (Ali et al., 2016; Kaiser et al., 2022; Snodgrass et al., 2017, 2023). Although such approaches often adapt a standardized mental health measure to local context, we opted to develop the SDRS from the ground up, based on prior research indicating that the extensive work required to adapt an existing resilience scale may not yield quantitatively meaningful or interpretable data (Mendenhall & Kim, 2019). While our results cannot speak to the relative strength of these alternative approaches to contextual scale development (given that we did not adapt the CD-RISC based on our qualitative data), our findings strengthen claims that cultural adaptation or local development of measures is essential for making subsequent quantitative data collection meaningful. Our findings also support the expansion of such a culturally-adapted scale development approach to strengths-based constructs, such as resilience, since the majority of the literature on adapting scales has occurred within the context of pathologic constructs, primarily depression and anxiety (Kaiser et al., 2013, 2019; Panter-Brick et al., 2018). Such an approach offers promising next steps for not only capturing mental health constructs in terms that are relevant to the population of interest, but also for shifting the focus to their strengths, rather than their deficits.

Our qualitative work similarly highlighted the value of taking a strengths-based approach that looks to communal networks, rather than individual traits. This is exemplified through the presence of themes of social support and a sense of religious connection, which go beyond the items more traditionally included in existing resilience scales. Such novel themes highlight the importance of looking at resilience among Somalis as extending beyond the individual. Prior research supports this hypothesis, finding that communal characteristics rather than individual ones are what drives resilience in this community (Frounfelker et al., 2019; Lincoln et al., 2016; Terrana et al., 2022). Frounfelker et al. (2019) have found that Somali community members are supported through both informal networks that offer social support and formal systems of mutual aid that provide opportunities for education and job training. Religiosity also emerged as a key source of comfort during trying times, serving as an additional bridge between the individual and the larger community of Somalis and Muslims alike (Frounfelker et al., 2019). Other investigations have similarly identified the importance of social bonds, both within the local community and to the larger Somali diaspora, providing a sense of belonging that buffers against the risks of social isolation (Lincoln et al., 2016). This shift away from conceptualizing resilience as an individual-level, trait-based construct is further supported by prior research that suggests communal-level, processual conceptualizations of resilience may provide more meaningful insight into other communities’ ability to withstand adversity (Kirmayer et al., 2011; Mendenhall & Kim, 2019; Terrana et al., 2022; Weaver & Kaiser, 2015).

This raises larger questions regarding how we might best measure resilience in situ and, more broadly, how to most responsibly and effectively engage in community-research partnerships to develop culturally tailored measures—particularly with regards to the feasibility of comprehensive validation of novel measures. While we aimed for ethnographic validity through qualitative methods via FGDs and cognitive interviewing, we also recognized a tension between generating a complete model that would have enabled us to psychometrically validate the SDRS and conducting a study that would be feasible for our community partner and community members. For example, given that resilience is theorized to serve as a moderator of the association between stressors and poor mental health, a complete model would require both exposure and outcome data, and our study lacked data on risk factors for negative mental health outcomes, such as past traumatic events or current day-to-day stressors (exposures) (Windle et al., 2011). Furthermore, the case for the SDRS's construct validity would be strengthened by its comparison with validated determinations of caseness for PTSD, anxiety, and depression among our participant population (Ellis et al., 2008, 2013; Terrana et al., 2022). In the absence of existing brief measures of these negative mental health outcomes that have been validated among Somali adults, the most appropriate alternative would have been to conduct clinical interviews with survey participants to establish a gold standard comparison for caseness, following the approach modeled by projects developing novel measures of mental health outcomes (Ali et al., 2016; Kaiser et al., 2022).

While the lack of comprehensive exposure data and validated determinations of caseness precludes a determination of the construct validity of the SDRS-R's construction of resilience as a moderator of good mental health, we carefully considered the trade-offs between collecting these data and the feasibility and acceptability of the associated data collection process within the Somali community. Performing comprehensive psychometric validation would have entailed not only recruiting a larger sample and placing a heavier survey burden on each participant, but also conducting clinical interviews. As such, we felt that it was important to prioritize participants’ stated desire to minimize survey burden and, instead, directed our attention towards the ways in which our approach might inform future efforts to develop localized measures in resource-limited settings. Such considerations are essential to building research-community partnerships that balance the needs of both parties to produce research products that are meaningful to the population of interest without placing undue burdens on individual community members.

While we hope that participants’ time investment towards producing a locally-contextualized measure translates into an instrument that generalizes across the Somali diaspora, we also recognize that drawing our sample exclusively from Somalis living in San Diego creates potential limitations to the generalizability of our findings and the SDRS's value among Somali communities elsewhere in the United States, in other countries, and in Somalia. While we suspect that the supportive constructs in the SDRS likely contribute to resilience among Somali community members worldwide due to their centrality to Somali culture, it is equally likely that local adaptations would strengthen the survey's applicability across settings based on contextual supports and stressors. This consideration is relevant not only among Somali communities, but within any community within which resilience depends on communal connections. While gathering individual level data can support the identification of community members who would benefit from proactive management to mitigate the development of psychiatric pathology, these data should be paired with efforts to gain a better understanding of the broader context of social, cultural, and environmental forces that inform individual-level outcomes (Hart et al., 2016; Weaver & Kaiser, 2015). Given the highly localized nature of these contextual factors, it is likely that the field will not only need to continue moving beyond the use of standardized measures that are intended to be applicable across diverse populations and cultural contexts, but also further develop resource-efficient protocols for creating scales that are both locally relevant and psychometrically sound.

### Study limitations

One of the primary limitations of this study is that the SDRS was only made available to participants in English. Although we had based this decision on our experiences in conducting FGDs, in which participants were comfortable discussing resilient themes exclusively in English, this may have resulted from the vast majority of FGD participants (86%) having already lived in the United States for more than five years. Given CHW reports that they assisted some participants with filling out the SDRS and other survey materials by providing translation services, it's clear that having a Somali version would be valuable, especially for newly arrived Somalis who do not speak English. We also did not differentiate between surveys that had been filled out exclusively in English and those that had been filled out with translation support from a CHW in our statistical models, which raises issues regarding the maintenance of equivalence of meaning across English and translated versions. Additionally, our decision to minimize survey burden limited our collection of robust data on past and current life stressors, which could have potentially served as a confounder of the relationship between resilience and poor mental health outcomes. Both translation into Somali and more comprehensive data collection that would allow us to examine associations between resilience and stressors/trauma represent invaluable next steps that would enhance our understanding of the SDRS’s utility as we continue to assess its psychometric validity.

Furthermore, our administration of the CD-RISC to a subsample six months after the initial measures of the SDRS-R, GAD, PHQ, and HTQ-PTSD for validation purposes may have undermined our comparison between the CD-RISC and the SDRS's associations with mental health symptoms. Finally, our decision to conduct cognitive interviews in a group setting, rather than as individualized sessions, deviates from the standard protocol for cognitive interviewing. While our approach essentially functioned as a highly structured focus group, this format may have impacted its utility.

## Conclusions

In our effort to identify the best indicators of resilience among Somalis through a rigorous qualitative analysis and culturally sensitive interpretations, we created a new measure of resilience, the Somali Distress and Resilience Scale (SDRS). This scale demonstrated more significant associations with measures of depression and PTSD than a standardized measure of resilience. This suggests its utility for identifying low resilience individuals who lack the support systems that might buffer them against the development of negative outcomes. It also demonstrates the complexity of measuring resilience using standard Euro-American-centric ideals and perspectives. Furthermore, the SDRS's development processes highlighted key considerations in partnering with communities to develop and comprehensively evaluate culturally contextualized measures of resilience. This points to the value of assessing resilience on multiple ecologic levels among communities in the Global South, as well as among diaspora communities, to advance this important strengths-based approach to addressing mental illnesses.

## Appendix 1. Preliminary Somali Resilience Survey items that were amended prior to finalizing the measure.

**Table:**

Survey item	Changes made
1. I feel obliged to support others when they are in need.	Amended to remove the reference to feeling a sense of obligation
2. There is at least one person in my life that I trust and can talk to during difficult or stressful times.	Amended to specify that this includes individuals other than God
3. There is at least one person in my life who can advise me when I have a problem.	
4. There is at least one person in my life who can offer me support in finding a job, housing, or transportation if I need it.	
5. Religious or spiritual beliefs are a source of strength for me.	Incorporated into unscored section on resilient supports due to consensus that Likert responses would likely adhere to cultural scripts
6. I feel a sense of oneness (*walaal-nimo*) with the broader Somali community.	
7. When I am having a difficult time, I have hope that tomorrow will be better.	
8. The Somali people are able to survive any challenges they face.	
9. No matter the challenges I face, I have the patience (*sabr*) to make it over and go on.	
10. I believe that things happen for a reason.	
11. I believe that God (*Allah*) will support me.	
12. I participate in organized religious activities.	
13. Prayer is an important part of my life.	
14. There are programs that are suitable to me as a member of the Somali community.	Incorporated into unscored section on barriers to good mental health to assess challenges to resilient outcomes
15. I have been able to find affordable housing in San Diego.	
16. Opportunities for schooling or job training in San Diego meet my needs.	
17. I am treated differently because of my race and my religion.	
18. I am able to see doctors who understand my unique needs as a Somali.	
19. I have difficulties with language barriers in my daily life.	
